# Abolishing Fees at Health Centers in the Context of Community Case Management of Malaria: What Effects on Treatment-Seeking Practices for Febrile Children in Rural Burkina Faso?

**DOI:** 10.1371/journal.pone.0141306

**Published:** 2015-10-26

**Authors:** Thomas Druetz, Federica Fregonese, Aristide Bado, Tieba Millogo, Seni Kouanda, Souleymane Diabaté, Slim Haddad

**Affiliations:** 1 School of Public Health, University of Montreal, 7101 avenue du Parc, Montréal, Québec, H3N 1X9, Canada; 2 University of Montreal Hospital Research Centre, 850 rue Saint-Denis, Montréal, Québec, H2X 0A9, Canada; 3 Biomedical and Public Health Department, Institut de Recherche en Sciences de la Santé, Ouagadougou 03 BP 7192, Burkina Faso; 4 Laval University Medical Research Center (CHUQ), Saint-Sacrement Hospital, 1050, chemin Sainte-Foy, Québec, Québec, G1S 4L8, Canada; 5 Faculty of Medicine, Laval University, 1050 avenue de la Médecine, Québec, Québec, G1V 0A6, Canada; University College London, UNITED KINGDOM

## Abstract

**Introduction:**

Burkina Faso started nationwide community case management of malaria (CCMm) in 2010. In 2011, health center user fees for children under five were abolished in some districts.

**Objective:**

To assess the effects of concurrent implementation of CCMm and user fees abolition on treatment-seeking practices for febrile children.

**Methods:**

This is a natural experiment conducted in the districts of Kaya (CCMm plus user fees abolition) and Zorgho (CCMm only). Registry data from 2005 to 2014 on visits for malaria were collected from all eight rural health centers in the study area. Annual household surveys were administered during malaria transmission season in 2011 and 2012 in 1,035 randomly selected rural households. Interrupted time series models were fitted for registry data and Fine and Gray’s competing risks models for survey data.

**Results:**

User fees abolition in Kaya significantly increased health center use by eligible children with malaria (incidence rate ratio for intercept change = 2.1, p <0.001). In 2011, in Kaya, likelihood of health center use for febrile children was three times higher and CHW use three times lower when caregivers knew services were free. Among the 421 children with fever in 2012, the delay before visiting a health center was significantly shorter in Kaya than in Zorgho (1.46 versus 1.79 days, p <0.05). Likelihood of visiting a health center on the first day of fever among households <2.5km or <5 km from a health center was two and three times higher in Kaya than in Zorgho, respectively (p <0.001).

**Conclusions:**

User fees abolition reduced visit delay for febrile children living close to health centers. It also increased demand for and use of health center for children with malaria. Concurrently, demand for CHWs’ services diminished. User fees abolition and CCMm should be coordinated to maximize prompt access to treatment in rural areas.

## Introduction

Malaria remains a major public health issue in Burkina Faso, where it causes ~40,000 deaths every year [1], most of them in children under five. Late and inappropriate treatment causes complications and results in increased lethality [2,3]. Yet if promptly administered, artemisinin-combination therapies (ACT) are effective to prevent death [4,5]. ACT treatment for children should be started as soon as possible after the onset of fever, preferably within the first 24 hours. In sub-Saharan African (SSA) countries, delays in seeking treatment from a recommended provider such as the primary healthcare center or a community health worker (CHW) are common and often due to the inadequate affordability, acceptability, and availability of healthcare resources and medications [6,7]. In Burkina Faso, it is estimated that only 27% of children under five are promptly and correctly treated [8]. Insufficient access to treatment is particularly salient in rural areas and among the poorest and least educated populations. Caregivers’ lack of education and information, poverty, and distance to the health center have been identified as common determinants of inappropriate treatment-seeking practices [9,10].

In 2010, national health authorities introduced community case management of malaria (CCMm) as a way to increase prompt access to ACT [11]. Across the country, one CHW per village was trained to administer ACT to febrile individuals. Visits to CHWs are free but the treatment costs ~0.4 USD. The initial objective of the national malaria program was that 80% of all uncomplicated malaria cases in rural areas would be managed by CHWs [8]. A progressive reduction in the proportion of malaria cases treated at health centers was expected. Simultaneously with the start of CCMm, a universal mass campaign of bed nets distribution was launched [12].

The CCMm strategy is now under implementation in many SSA countries [13]. In some settings, after its introduction, treatment coverage for malaria was improved–notably in remote areas–and health centers’ workload was reduced [14–17]. However, CCMm has encountered difficulties in its implementation in Burkina Faso, such as recurrent drug stock-outs; a lack of adequate training, remuneration and supervision of CHWs; and poor participation of communities [18,19]. Studies have shown that the use of CHWs is still very limited, even in rural areas [20,21].

In the meantime, non-governmental organizations (NGO) have tried in several health districts to improve financial access to primary healthcare centers by removing user fees. Studies have shown that abolishing user fees increases the use of health centers and improves access to treatment, including among children presenting an episode of malaria [22–26]. The present study was conducted in one of the health districts (Kaya) where user fees have been abolished. The abolition was initiated in 2011 for all children under five visiting healthcare facilities and is part of a broader intervention implemented by the NGO Save the Children. The intervention only affects health centers, not CHWs, such that user fees still apply for antimalarial treatments provided to children by CHWs.

This study focuses on the combined effects of user fees abolition and CCMm. While the outcomes of these two interventions have been studied in various settings, little is known about the effects of their concurrent implementation. A recent study in Sierra Leone showed that superimposing CCMm on free healthcare did not improve treatment-seeking practices for malaria, likely because of a non-strategic deployment of CHWs [17]. The context of the present study presents the reverse situation, as user fees abolition was superimposed on CCMm. The study’s main objective was to assess the contribution of user fees abolition to the improvement of treatment-seeking practices in a context of nation-wide CCMm. The main hypothesis of the present study was that the abolition of user fees would (1) increase the likelihood that a febrile child would be brought by his/her caregiver to a health center and (2) shorten the time elapsed before visiting a health center. A secondary hypothesis to be validated was that, while CCMm alone would not affect the use of primary healthcare centers, the superimposed abolition of user fees would led to increased use.

## Methodology

### Setting

This study was part of a research program initiated in 2010 to study the effects of malaria-related interventions on child morbidity in Burkina Faso. The study was conducted in two health districts of Burkina Faso (Kaya and Zorgho) that are similar in terms of composition, population size, poverty level, and provision of healthcare services [18]. Climate and malaria transmission rates are also comparable. In both districts, malaria is holo-endemic and characterized by a season of high transmission from June until November.

CCMm was implemented in both sites in late 2010. User fees were abolished in Kaya in July 2011, but only for children under five, who are eligible for free care at health centers. User fees were not abolished in Zorgho, which was used as a comparator for some analyses. The study area covers a rural zone situated within a 20-km radius from the districts’ main cities. Details of the selection and description of study sites have been presented elsewhere [19,27].

### Study design

The study was a natural experiment [28]. The effects of the interventions were evaluated under real-world conditions of implementation and were compared between the intervention (Kaya) and control (Zorgho) districts. Longitudinal and cross-sectional data were collected over ten years, both retrospectively (2005–2011) and prospectively (2011–2014). The design combined three components.

The first was a pooled time series using administrative data collected from rural health centers in both sites. Its aim was to assess changes in the use of primary healthcare services by children with malaria: (1) after user fees were abolished in the intervention district of Kaya; and (2) after CCMm was introduced in the control district of Zorgho–but not in Kaya, because user fees were abolished too soon after CCMm was implemented. The outcome of interest was the monthly number of visits of children who received a diagnosis of malaria at each health centre.

The second component used data from a population-based cross-sectional survey conducted in Kaya less than one month after the abolition of user fees, i.e., in August 2011, during the peak of malaria transmission. Its aim was to evaluate this intervention’s effects on treatment-seeking practices for children with a suspected episode of malaria. Because of the lack of any pre-intervention measure, the contribution of user fees abolition was estimated by comparing treatment-seeking practices between households where the caregiver knew user fees had been abolished and households where the caregiver did not know.

The third component used data from a population-based cross-sectional survey conducted in Kaya and Zorgho one year later, during the 2012 peak of malaria transmission (in August again). Its aim was to evaluate the effects of user fees abolition on time elapsed before visiting primary healthcare centers. The contribution of user fees abolition was estimated by comparing time elapsed before visits between the two sites.

### Data collection

Data were collected from two different sources: registers of primary healthcare centers and a panel study of households conducted in the study area.

*Administrative data* (design component 1) were collected from the registers of all primary healthcare centers covering the study area (four in Kaya, four in Zorgho). The number of visits of children under 14 years of age was aggregated by month and categorized according to age (<5 or 5–14) and diagnosis (malaria or other). Data aggregation and entry were double-checked. The observation period spanned from January 2005 to June 2014 (i.e., 114 observation months). The size of the population in each health center’s catchment area was obtained from official statistics.

*Population-based data* (design components 2 and 3) were extracted from a larger longitudinal study conducted in a panel of 2,232 randomly selected households in the two districts [20]. Households in Kaya were extracted from the Kaya Health and Demographic Surveillance System database [27]. For the purposes of this study, only households from rural areas were used in the analyses (684 in Kaya, 351 in Zorgho; total n = 1035), since CHWs are not used to treat malaria in urban areas [20]. Households were surveyed once a year during the peak of malaria transmission. All children under five were followed; a survey was administered every year to document their health status, recent febrile episodes (within a two-week recall period), and treatment-seeking practices. Questionnaires were adapted from the Malaria Indicator Survey [29]. All households agreed to participate to the study. Losses to follow-up were replaced by random selection from the same district. The main characteristics of the households and children involved in this study are presented in Table 1.

**Table 1:** Characteristics of participating households and children.

		2011	2012	
		Kaya	Kaya	Zorgho
**Households**				
Number		684	707	376
Median size		7	7	10
Know about user fees abolition		87%	100%	/
Own at least 1 cellphone		NA	83%	85%
Usually bikes or walks to the health center		72%	72%	78%
Distance to health center	<2.5 km	57%	57%	52%
	2.5–5 km	30%	29%	31%
	>5 km	13%	14%	17%
**Children <5 years**				
Number		1073	1126	787
Median age, in months (interquartile range)		31 (26)	32 (27)	32 (28)
Female		50%	50%	50%
Slept under a bednet the night before		67%	94%	95%
Two weeks before interview were:	Febrile	24%	23%	20%
	Febrile and with signs of severity	7%	7%	4%

### Analysis

Data from the health centers’ registers were analyzed using an interrupted time series approach focused on changes in intercept and slopes after introduction of the intervention. The negative binomial link function was used in the statistical models. Trends, seasonality, size of catchment area (exposure variable), and the hierarchical and longitudinal structure of the data were taken into consideration [30,31]. Intervention effects were estimated by incidence rate ratios (IRRs). Applying the triple difference approach, a ratio of IRRs was calculated to improve the robustness of the estimated effects of user fees abolition [32,33]. Three combined counterfactuals were used: (1) the pre-intervention period in the intervention site (Kaya); (2) the control site (Zorgho), where user fees remained in place; and (3) children 5–14 years of age who were not eligible for user fees abolition.

Population-based data were analyzed using multinomial or competing risks models, depending on the nature of the outcome variable. In the former case, the dependent variable was the first reported treatment action taken by the caregiver for a febrile child. Responses fell into one of four categories: self-medication, visit to a CHW, visit to a health center, or no action. The independent variable was the caregiver’s knowledge (yes/no) that fees had been abolished. The model was adjusted for clustering in households and for child and family confounders (sex, age, fever duration, presence of danger signs, use of a bed net the night before, household size, socio-economic status, distance to the nearest health center).

Fine and Gray’s adaptation of the Cox model [34] was used to evaluate the effects on time elapsed–expressed in days, as counting based on 24 hours was not possible [35]–before visiting a health center. Three categories of febrile children were defined: those who visited a health center, those who used a “competitor” (CHW, self-medication), and those who received no treatment. Models were adjusted for clustering and confounders (as described above). The predicted hazard ratio of visiting a health center on Day 1 of fever was computed by estimating a time-dependent covariate.

### Ethical approvals

Ethical approvals were obtained from both the research ethics committee of the University of Montreal Hospital Research Center in Canada and Burkina Faso’s health research committee. Data were used in conformity with Kaya Health and Demographic Surveillance System policy (authorization 1KH001-2015). Written informed consent was obtained every year from every respondent and from the caretakers of children on their behalf. Children with danger signs were immediately referred to a health center.

## Results

### Use of primary healthcare centers by children with malaria

**Fig 1:**
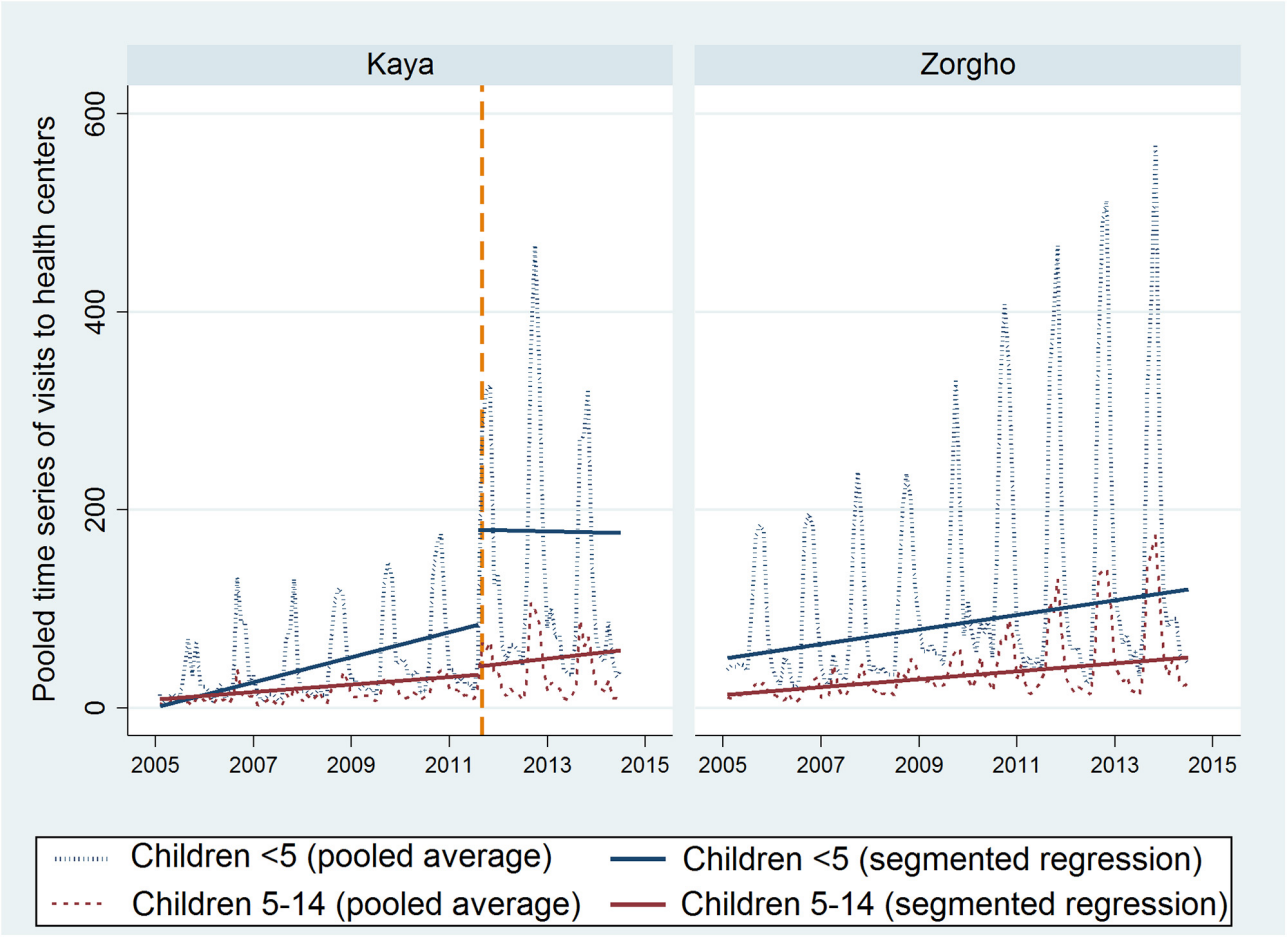
Average number of visits to Health Centers by month (children <14 years with a diagnosis of malaria).

The average monthly number of visits of children with malaria ranged from 0 to 600, depending on the year, the season, the age group, and the district (Fig 1). Pooled interrupted time series demonstrated a secular trend in both sites during the time frame, as well as important seasonal variations. There was no significant difference in the use of health centers in the district of Zorgho after the introduction of CCMm, neither immediately (IRR for intercept change = 0.88; p = 0.456), nor during the two-year period that followed.

In contrast, the abolition of user fees in Kaya significantly increased the use of health centers for children under five with malaria (IRR for intercept change = 2.1; p <0.0001) (Table 2). No change was observed for non-eligible children, i.e., those 5–14 years old (IRR = 1.26; p = 0.464). The significant ratio between eligible and non-eligible children confirms the robustness of these results (ratio of IRRs = 1.69, p = 0.001).

**Table 2:** Predicted number of visits to health centers by children with malaria (across districts and age groups, 2005–2014).

Time = 79^¶^	Kaya			Zorgho		
	Before	After	IRR	Before	After	IRR
	(UFA = 0)	(UFA = 1)	(CI 95%)	(UFA = 0)	(UFA = 0)	
**Children <5**	84.42	180.26	2.13***	91.68	91.68	1
			(1.55–2.93)			
**Children 5**–**14**	33.72	42.56	1.26	33.98	33.98	1
			(0.68–2.36)			
			**Ratio of IRRs**			
**Kaya vs. Zorgho, after vs. before,**			1.69**			
**children <5 vs. 5**–**14**			(1.24–2.29)			

Notes: UFA: user fees abolition (0 = No; 1 = Yes)

IRR: incidence rate ratio

CI: confidence interval

**p <0.01

***p <0.001

^¶^Predicted values at the time of UFA introduction; negative binomial model is adjusted for monthly variation.

### Effects of user fees abolition on treatment-seeking practices

**Fig 2:**
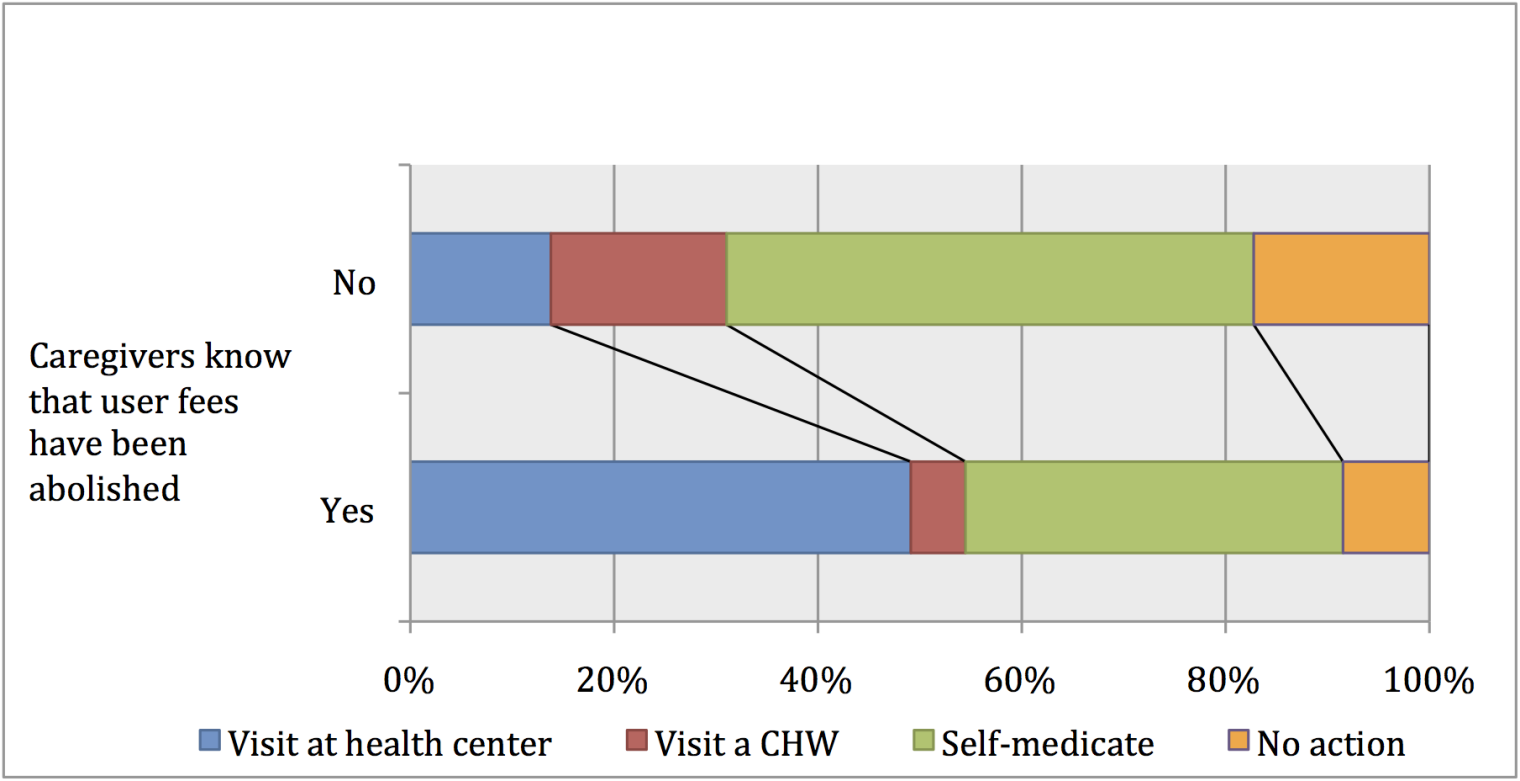
Treatment-seeking practices for febrile children according to the caregivers’ knowledge about the abolition of user fees (Kaya, 2011).

In Kaya district, 253 (24%) children under five were reported febrile in the two weeks prior to the 2011 survey. Among these children, there were marked differences in treatment-seeking practices depending on whether the caregiver had heard about the recent abolition of user fees (Fig 2). The statistical model confirmed these differences. The likelihood of bringing a febrile child to a health center as a first recourse was three times higher (risk ratio (RR) = 3.12, p < 0.001) when the caregiver knew the services were free (Table 3). The use of CHWs mirrored this result (RR = 0.33; p <0.05). Finally, the likelihood of not seeking any treatment was reduced by more than half (RR = 0.44; p <0.05).

**Table 3:** Predicted probabilities of treatment-seeking practices for febrile children (Kaya, 2011).

	Caregivers know that user fees have been abolished^¶^		Risk difference	Risk ratio
	No	Yes	(95% CI)	(95% CI)
**First action**	(A)	(B)	(B-A)	(B/A)
Health center	0.16	0.49	+ 0.33***	3.12*
			(0.18 to 0.48)	(1.27 to 7.64)
CHW	0.16	0.05	- 0.11	0.33*
			(-0.24 to 0.03)	(0.12 to 0.92)
Self-medication	0.49	0.38	- 0.12	0.76
			(-0.31 to 0.08)	(0.5 to 1.16)
No action	0.19	0.08	- 0.11	0.44*
			(-0.24 to 0.03)	(0.2 to 0.98)

Notes:

CI: confidence interval

HC: health center

*p <0.05

***p <0.001

^¶^Multinomial model adjusted for household-level variables (SES, distance to HC), individual-level variables (sex, duration of fever, severity signs, slept under a bednet) and family clustering.

In the two weeks prior to the 2012 survey, 421 children were reported febrile: 260 (23%) in Kaya and 161 (20%) in Zorgho. In the subsample of children brought to a health center, the average time elapsed was significantly shorter in Kaya than in Zorgho (1.46 days in Kaya vs. 1.79 days in Zorgho; p <0.001). The competing risks model revealed that the adjusted hazard ratio (the ratio of instantaneous likelihoods) of visiting a health center was significantly higher (p = 0.03) for Kaya households living close to the health center (<5 km), which means the time elapsed before the visit was significantly shorter than for their counterparts in Zorgho [36]. Hazard ratios also significantly decreased as household size increased (p = 0.02).

**Fig 3:**
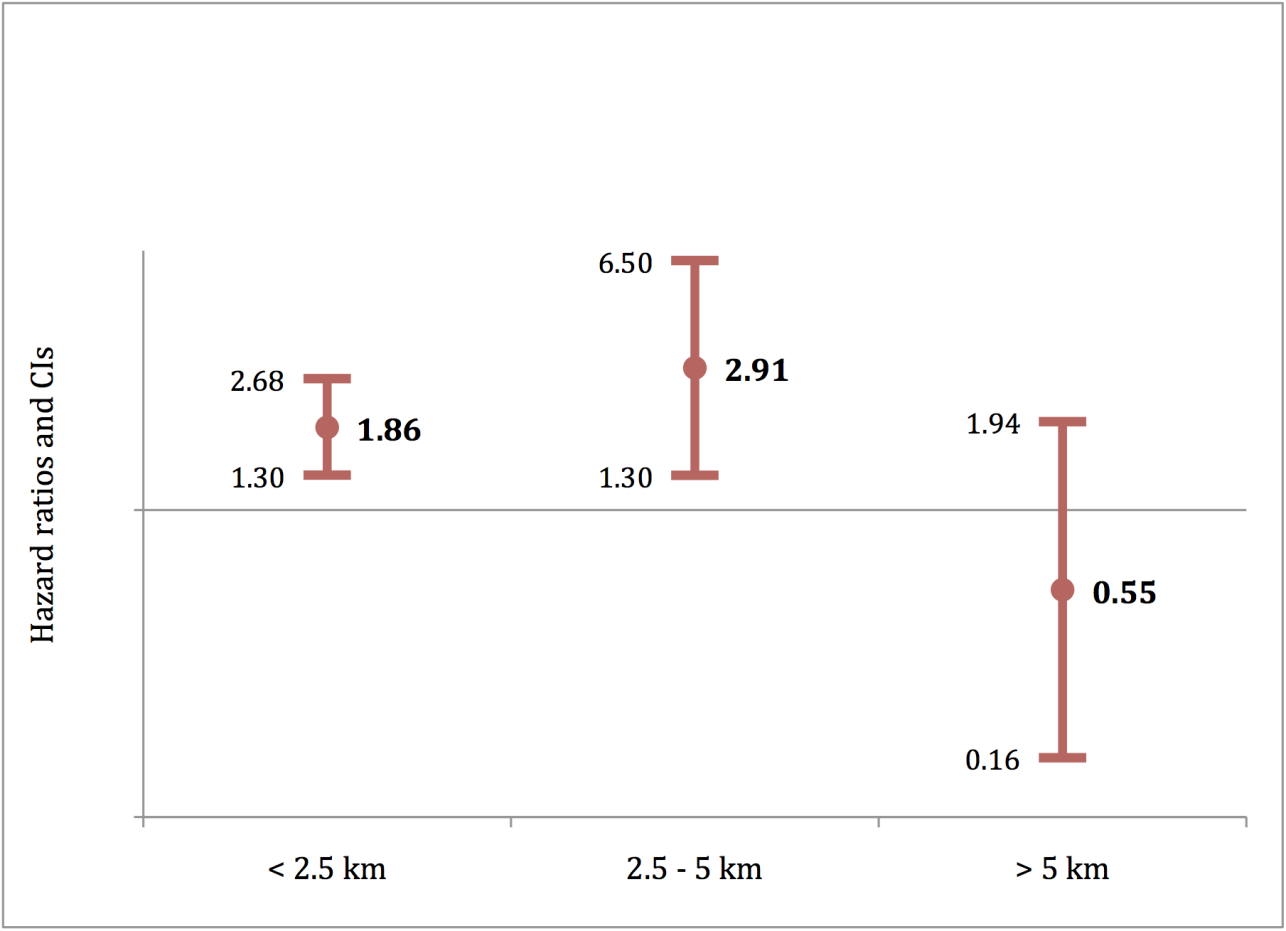
Instantaneous likelihood of visiting a health center on Day 1 of fever for children <5 in Kaya vs. Zorgho according to household distance from center (2012).

Predicted hazard ratios of visiting a health center on Day 1 of fever were computed according to the distance between household and health center (Fig 3). At Day 1, the instantaneous likelihood of visiting a health center is nearly two times higher for households living close to a health center (<2.5 km), and nearly three times higher for households living between 2.5 and 5 km from the health center. There was no significant difference for households living far from a health center (>5 km).

## Discussion

The abolition of user fees had beneficial effects on treatment-seeking practices. First, it was associated with increased use of health centers, reduced absence of treatment-seeking actions, and reduced use of self-medication (although the latter was not statistically significant). Second, abolishing user fees may have increased the promptness of visits to a health center in cases of fever in children; to our knowledge, this is the first study indicating effects on promptness. This result is consistent with studies showing that time elapsed before treatment of obstetric complications was reduced by the abolition of user fees for pregnant women [37]. By increasing caregivers’ propensity to bring their febrile children to health centers and reducing the associated delay, the abolition of user fees may contribute to decreasing malaria-attributable mortality [38,39].

However, the positive effects of user fees abolition on promptness of visits were only observed for households living relatively close to a health center (<5 km). For households living further away, the use of CHWs in villages may still be a worthwhile option. Indeed, CHWs’ treatment coverage is higher in remote areas with no health center in proximity [20,40,41]. Also, caregivers usually visit them promptly–84% of visits to CHWs occurred within 24 hours in this study, and 70% in that of Rutebemberwa et al. [35]. CHWs could be most useful in remote areas with difficult access to health centers.

Regarding the effects of CCMm on the healthcare centers’ workload, this study found no evidence that this program had any incidence on the use of primary healthcare centers by children with malaria. This result differs from what other studies have reported. In an interventional study conducted in Burkina Faso, Tiono et al. observed a significant reduction in the use of health centers by febrile children [16]. In a trial conducted in Zambia, Seidenberg et al. observed a significant reduction in the likelihood of febrile children being brought to health centers [42]. However, these studies did not perform long-term or time-series analyses. In addition, an important feature of the present study is that CCMm was evaluated under real-world conditions of implementation. Estimates reflected its practical effectiveness rather than its efficacy. The absence of noticeable impact on health center use is consistent with the results of previous studies in that country that highlighted febrile individuals’ low propensity to visit CHWs [20,21,43].

Even in the presence of a CCMm strategy, convergent evidence from the population-based and administrative components of our analyses shows there was a shift in treatment-seeking practices after the abolition of user fees. First, the demand for services at primary healthcare centers increased considerably. This evidence is bolstered by the fact that healthcare center use increased only among eligible children. While several studies have observed higher volumes of consultations at health centers after the abolition of user fees for children [22,25,44,45], this study examined this effect in parallel with a higher demand for healthcare services to treat malaria. Arguably, even though CHWs geographical access is better, primary healthcare centers may nevertheless attract a large part of the population due to the abolition of user fees.

On the other hand, the shift also entailed a significant decrease in the likelihood that a febrile child would visit a CHW; this was an unanticipated effect of the abolition of user fees. The fact that caregivers have to pay when they bring their children to a CHW, whereas they can bring them to a qualified nurse for free, has likely contributed to this substitution effect, as suggested by a previous qualitative study on CHWs’ performance [19]. Insufficient coordination and health services fragmentation have been acknowledged as important issues [46,47], especially in SSA, due to the multiplicity of health actors [48]. Governance issues are partly due to national health authorities’ limited capacities to negotiate the terms of interventions introduced by large NGOs. The lack of sustainability is related to this asymmetric relationship, with countless instances of national authorities terminating interventions for which NGO funding had been withdrawn. In the present context, this is of great concern because a change in treatment-seeking practices induced by the abolition of user fees could raise issues for community programs in the future. Should the user fees abolition end, CHWs could arguably encounter difficulties in renewing the confidence and interest of community members–confused about which healthcare provider to visit in case of fever. Access to care in remote and hard-to-reach areas (where CHWs are the most helpful) might therefore be undermined in the long-term. This illustrates the importance of coordinating interventions and addressing the issue of their sustainability before their implementation.

While health governance problems are often debated at the global or national levels [49], this study has revealed empirically that local and concomitant interventions might be detrimental to each other if they are not properly interconnected. Lack of coordination is not the only implementation issue that CCMm faces [50–53], but it seems to have further undermined its effectiveness in Burkina Faso [19,21]. In the same vein, there is arguably a lack of coherence among official strategies against malaria. While many efforts are being made in Burkina Faso to improve primary healthcare services and their coverage [54], CCMm sets up a second community-based system in all settings of the country [55], and not only in the remote and underserved areas for which it was intended [11]. Strong leadership of health authorities at the district level is needed to cope with these issues of coordination among interventions and among actors [56].

### Limitations

Study sites were not randomly assigned or selected. The control site was chosen because it shared many features with the intervention site in terms of the most important confounders. However, there may have been unobserved sources of heterogeneity between the sites. To reduce selection bias and increase the internal validity of the conclusions, several methods were used: juxtaposition of several quasi-experimental designs [30], replication of the same hypothesis on several outcomes (theoretical replication), and the use of several counterfactuals (literal replication) [57]. When comparing treatment-seeking practices between caregivers who knew about the user fees abolition and those who did not, reverse causation could not be ruled out; caregivers might have known because they visited a health center, rather than having visited a health center because they knew. Finally, the study area is located in the countryside but is relatively close (<20 km) to medium-sized cities, which should be taken into consideration for external validity. The effects of the user fees abolition may have been different in more remote areas, even if no informal interviews or local collaborators suggested this.

## Conclusion

This study showed that abolishing user fees at health centers contributed to improving treatment-seeking practices for children with malaria in a rural context where CCMm was already present. The intervention increased febrile children’s likelihood of being brought to a health center and was associated with shorter times before visits.

The abolition of user fees also had the unanticipated effect of reducing children’s likelihood of visiting a CHW in cases of fever. The multiplicity of health stakeholders at the local level, a lack of strong leadership in the coordination of the two interventions–the abolition of user fees and CCMm–and deficiencies in CCMm planning at the national level are all likely to have contributed to this situation. Geographical and financial barriers remain the greatest impediments to healthcare access in rural Burkina Faso. Eliminating these barriers will require a clearly articulated and coordinated strategy [58].

## Abbreviations

ACT: Artemisinin-combination therapy
CCMm: Community case management of malaria
CHW: Community health worker
IRR: Incidence rate ratio
NGO: Non-governmental organization
RR: Risk ratio
SSA: Sub-Saharan Africa
